# Applying an authentic partnership approach to facilitate optimal health of Aboriginal children

**DOI:** 10.1017/S1463423622000329

**Published:** 2022-08-15

**Authors:** Naomi Sprigg dos Santos, Garth Kendall, Ailsa Munns

**Affiliations:** 1 Clinical Nurse Manager, Goldfields Population Health, Leonora, Western Australia, Australia; 2 School of Nursing, Midwifery and Paramedicine Faculty of Health Sciences, Curtin University, Perth, Western Australia, Australia; 3 Course Coordinator Child and Adolescent Health Programs, Coordinator Community Mothers Program (WA)- School of Nursing, Midwifery and Paramedicine Curtin University, Perth, Western Australia, Australia

**Keywords:** partnership approach, aboriginal, early childhood, cultural sensitivity, social determinants, interpersonal relationships, mutual goal setting

## Abstract

The aim of this development paper is to inform the ongoing implementation of the partnership approach with Aboriginal families in Australia. As almost all Community Health Nurses employed by the Health Department of Western Australia, Country Health Service are non-Aboriginal, there are a number of factors that may, potentially, limit their capacity to work effectively with the primary caregivers of Aboriginal children.

Historically, much that has been written about the health and development of Aboriginal people in Australia has been negative and derogatory with wide criticism for their non-participation with health services and healthy lifestyle activities. Not only has this “deficit discourse” approach proved to be unhelpful in terms of improving the health and well-being of Aboriginal people but also there is mounting evidence that it has been detrimental to mental and physical health and capacity to achieve autonomy in all aspects of life.

In response to the voices of Aboriginal people, the partnership approach to care has been promoted for use by Community Health Nurses in Western Australia. However, the implementation of the approach is not always genuinely strength based, and it does not always focus on mutual goal setting within authentic partnership relationships. The partnership approach has the potential to improve the lives of Aboriginal people if it is implemented with appropriate cultural sensitivity, shared responsibility, dignity and respect.

## Introduction

The early years of life are of critical importance for health and well-being across the life course (Kelly, 2018). There is increasing recognition of the importance of supporting women during pregnancy and families with young children (Moore *et al.*, 2017). The partnership approach to care has been widely promoted for use by Community Health Nurses (CHN) working within the Western Australian Country Health Service (WACHS) (Department of Health, 2017; Munns *et al.*, 2018), and it is currently applied to facilitate the optimal health and well-being of preschool-aged Aboriginal children. The approach should be strength based and focus on interpersonal relationships and mutual goal setting (Keatinge *et al.*, 2008). While the partnership approach has great potential, it is questionable whether all CHN currently have the knowledge, attitudes and skills required to adopt it effectively with Aboriginal families (Scougall, 2006; Bainbridge *et al.*, 2015; Munns *et al.*, 2018). The successful implementation of the approach, as with all programs designed to benefit Aboriginal people, requires that nurses are culturally competent (Bainbridge *et al.*, 2015). In a recent ‘Closing the Gap’ document, Bainbridge *et al.* (2015: 3) state that cultural awareness training, alone, is not sufficient to prepare non-Aboriginal staff to work effectively in the delivery of health services for Aboriginal people. As almost all CHN employed in Western Australia (WA) are non-Aboriginal (Durey *et al.*, 2016a), there are a number of factors that may, potentially, limit their capacity to work effectively with the primary caregivers of Aboriginal preschool-aged children. First, non-Aboriginal people often have limited understanding of the positive aspects of Aboriginal culture and family life (Fogarty *et al.*, 2018b). Second, while attitudes toward Aboriginal people in Australia have improved in recent years, there is still considerable progress that needs to be made (Osbourne *et al.*, 2013; Fogarty *et al.*, 2018b). Third, there is evidence that non-Aboriginal health workers frequently lack key skills required to facilitate the necessary behaviour changes (Bainbridge *et al.*, 2015). More specifically, many non-Aboriginal people have considerable difficulty communicating openly with Aboriginal people and setting mutual goals, (Durey *et al.*, 2016a; Lin *et al.*, 2016; Munns *et al.*, 2017), and they do not always consider the physical and psychosocial contexts of Aboriginal people’s lives (Stevenson *et al.*, 2017; Fogarty *et al.*, 2018a).

The aim of this development paper is to inform the ongoing implementation of the partnership approach with Aboriginal families in Australia. We begin by outlining the partnership approach to care and the implementation of the approach with Aboriginal families in WA. Next, we emphasise that social factors, not Aboriginality per se, determine health and developmental outcomes for Aboriginal children. We then draw attention to the importance of understanding how historical events have shaped the lives of Aboriginal people and how the “deficit discourse” has blinded non-Aboriginal people to the many positive aspects of Aboriginal culture and family life. Having identified the key issues, we then explain how Aboriginal people have become united in their voice for change and we outline an authentic partnership approach for the delivery of services.

## A partnership approach, but a lack of shared goals

The partnership approach to care has been adopted in Australia and internationally as an approach that promotes the engagement and participation of families (Splaine Wiggins, 2008; Department of Health, 2017). In the WA Aboriginal Health & Wellbeing Framework 2015–2030 partnership is one of the guiding principles to improve the health and well-being of Aboriginal people (Department of Health, 2017). The focus of the approach is on the relationship between the health professional, client and family. The relationship should be strength based and one of equality and acknowledgement of each party’s expertise and competence (Splaine Wiggins, 2008). The health care provider has knowledge of health information, and the client understands their and their family’s life situation and intentions. Trust needs to develop so that clients and health care providers can discuss their concerns and limitations openly and to set attainable goals (Cann and Gardner, 2012). The mutually established goals are more likely to be realistic because they have considered the viewpoints and constraints of the stakeholders. From the client’s perspective, the partnership approach enhances the sense of empowerment and contributes to an increase in self-efficacy and self-esteem (Splaine Wiggins, 2008).

When Aboriginal primary caregivers bring their preschool-aged child to see a CHN, it is usually with a specific purpose in mind. Common reasons for attendance include symptoms of coughs and colds, gastrointestinal upsets, parasitic infestations, skin lesions and infections and middle ear infections. For the health professional involved, this presentation offers the opportunity to assess a wide range of health and developmental factors, including: iron deficiency; immunisation; physical growth; speech and language development; fine and gross motor skills; emotional, attentional and social regulation and problem solving skills. Both the primary caregiver and health professional may hope to achieve one or more goals. For the primary caregiver, it is a solution to the concern, while for the health professional, it is to identify health and developmental issues and to either enhance the primary caregiver’s capacity to manage the problem or refer to specialist care. This difference in goals can contribute to frustration, and most importantly, the failure to address the needs of one, or both parties. The lack of shared goals can be one of the greatest barriers to the engagement of health services with Aboriginal families and the collaborative achievement of optimal health and developmental outcomes for Aboriginal children (Durey *et al.*, 2016a). Arguably, the biggest barrier to mutual goal setting is the lack of synergy of health and well-being priorities between the CHN and Aboriginal people. Aboriginal people traditionally view health in terms of social and emotional well-being. This acknowledges the importance of connection to the land, spirit, culture, family and community. CHNs who are working from a medical model will fail to set mutual goals with Aboriginal clients and families until they can work from the social and emotional well-being ethos (Department of Health, 2017). Despite the wide implementation of the partnership approach to care, it is questionable whether it has been implemented effectively with Aboriginal families (Bainbridge *et al.*, 2015).

## Social factors determine health and developmental outcomes for aboriginal children

There have been improvements in the health and developmental outcomes of Aboriginal children in Australia (Alsop-Shields and Dugdale, 1995; Gracey, 1998; Fogarty *et al.*, 2018a). However, Aboriginal Australians continue to live with poorer health than non-Aboriginal Australians (Hunt, 2013). The life expectancy of Aboriginal people is approximately 10 years less than for non-Aboriginal Australians (Anderson *et al.*, 2018). Despite improvements in some areas, there is resounding evidence that Aboriginal children experience worse health and developmental outcomes than non-Aboriginal children (Arefadib and Moore, 2017). The health of preschool-aged children living in very remote areas is poorer than the health of preschool-aged children living in less remote or metropolitan areas. This is noteworthy as Western Australia is a vast state with much of its area classified as being either remote or very remote (Anderson *et al.*, 2018).

The health and well-being of children is shaped by the social and economic conditions in which they live (Osbourne *et al.*, 2013). Many Aboriginal children in Australia are growing up in families that have insufficient financial resources (Dockery *et al.*, 2010; Taylor and Edwards, 2012). Compared with non-Aboriginal families, Aboriginal families have fewer employment opportunities and lower levels of income (Zubrick and Silburn, 2006; Dockery *et al.*, 2010; Taylor and Edwards, 2012). Lower educational attainment often results in greater difficulty in obtaining employment and lower wages for those who are employed (Zubrick and Silburn, 2006). Unemployment and low income then lead to poor housing and food insecurity (Zubrick and Silburn, 2006; Dockery *et al.*, 2010; Taylor and Edwards, 2012). There is considerable evidence that stressful life events in the family effect the trajectory of a child’s life (Kendall *et al.*, 2009). The authors of research undertaken using data collected in the Western Australian Pregnancy Cohort (Raine) Study have found that the exposure to stressful life-events, such as financial difficulties, unemployment and dysfunctional relationships, negatively impacts children’s behaviour and mental health (Robinson *et al.*, 2008). Many Aboriginal children are exposed to multiple family stressful life-events. For example, on average Aboriginal children are more likely than non-Aboriginal children to be exposed to family violence and substance abuse. The long-term consequences to health and well-being are far reaching (Kendall *et al.*, 2009). This phenomenon has become known as biological embedding (Vélez-Agosto *et al.*, 2017). Biological embedding refers to the impact of a child’s environment and experiences on their genetic predisposition and neuroendocrineimmune response to stress (Kendall *et al.*, 2009). The process of biological embedding is implicit in the Bioecological Model of human development that highlights the importance of a child’s environment, both proximal and distal, on development, health and well-being (Dockery *et al.*, 2010). Security in food, safe housing and adequate financial resources afford people a stronger sense of control and a decrease in their levels of stress (Dockery *et al.*, 2010; Taylor and Edwards, 2012). This has been demonstrated to improve educational, behavioural and health outcomes for children which, in turn, leads to improved health outcomes for adults (Shonkoff *et al.*, 2009; Maggi *et al.*, 2010).

## Historical events and the deficit discourse

In order to understand the social determinants of Aboriginal health, it is important to be aware of the impact of colonisation. Historically, the rights, culture and knowledge of Aboriginal people were disregarded. Since 1770 when Captain James Cook proclaimed the land to be ‘terra nullius’, or nobody’s land, (Osbourne *et al.*, 2013) Aboriginal people have been struggling to survive. Colonisation has been a devastating process that has spanned many generations of Aboriginal people (Sherwood, 2013). From colonisation in 1788 until the census of 1967 Aboriginal people were considered by law to be part of the ‘fauna’ of Australia (Osbourne *et al.*, 2013). In the 1800s, many Europeans living in Australia believed that the Aboriginal population would be eliminated. However, when it became apparent that Aboriginal people had survived the initial brutal impact of colonisation government policies to control and segregate Aboriginal people were established. In Western Australia, policies were developed to this effect in 1886 with the Aborigines Protection Act and Aborigines Act of 1905 (Sherwood, 2013). In 1915, the newly appointed Protector in Western Australia, Mr Neville, ensured that oppression was heightened as further control was given to the government over Aboriginal people with the implementation of the 1936 Aborigines Act Amendment Act (Houston, 2000). This coincided with the depression of 1929 which only augmented the already severely harsh circumstances for Aboriginal people (Van den Berg, 1995; Houston, 2000). These laws had a shattering impact on Aboriginal people across the state (Dudgeon and Walker, 2015). The 1936 Act extended the definition of “Native” to include almost all Aboriginal people in the state. The guardianship of “Native” children was allocated to the Protector and the age was raised from 16 years to 21 years of age, this was regardless of whether the parents were married or not. Non-Aboriginal people wishing to employ Aboriginal people had to apply for permits which made their employment difficult (Houston, 2000). The Protector also had the power to deny permission for an Aboriginal person to marry (Van den Berg, 1995). The government of Western Australia then implemented the 1944 Citizenship Act. This meant that Aboriginal people could apply to the “Protector of Natives” for an exemption to the oppression of the 1936 Act under the conditions they did not associate with “Natives” or speak their language (Van den Berg, 1995; Houston, 2000). By this time many Aboriginal children had been forcibly removed from their families and were being raised in institutions most often with their whereabouts unknown to their parents and no contact (Arndt, 2017). Many Aboriginal families believed that applying for the exemption under the 1944 Citizenship Act would give them the rights to have their children returned. Tragically this was not the case (Sherwood, 2013). The 1944 Citizenships Act also allowed Aboriginal people to purchase and consume alcohol; however, it remained illegal for them to share the alcohol with those who had not applied for the exemption (Osbourne *et al.*, 2013). As land access for Aboriginal people became restricted so did the ability to hunt and continue cultural practices that had been in existence for thousands of years. Many Aboriginal people were suffering from malnutrition and poor health. In order to survive it was common for Aboriginal people to work for farmers and station owners in return for food rations (Sheehan, 2012). In the census of 1967 Aboriginal people were counted for the first time. This was seen as a symbolic acknowledgement that Aboriginal people were the first Australians (Osbourne *et al.*, 2013). It took some years, however, to end the policies of assimilation which included children being forcibly removed from their mothers (Sheehan, 2012). The Native Title Act of 1993 was a major breakthrough in the recognition and protection of native title. It allowed for a framework around establishing where and with whom native title exists and determined compensation when native title is effected by other Acts. The 1990s was a decade which saw the recognition that many policies and practices were not addressing the social, economic and health disadvantages facing Aboriginal people. In 2008, the Australian Government made a formal apology to the Aboriginal and Torres Strait Islander people. Following the apology, the Council of Australian Governments (COAG) established a set of strategies to address the social, economic and health disadvantages experienced by many Aboriginal Australians. These have become known as the Closing the Gap (Osbourne *et al.*, 2013).

Much that has been written about the health and development of Aboriginal people in Australia over many years has been negative and derogatory (Fogarty *et al.*, 2018b). Aboriginal people have been widely criticised for not participating in available health services and engaging in lifestyle promoting activities (Dudgeon *et al.*, 2014; Fogarty *et al.*, 2018a). Similarly, Aboriginal parents and primary caregivers have been criticised for not adopting mainstream child-rearing practices and optimising their children’s health and developmental outcomes within a non-Aboriginal context (Fogarty *et al.*, 2018a). According to ‘Arnsteins Ladder’, the amount of involvement and engagement a person or people group has within the society in which they live is directly proportionate to how that society views and treats them. Aboriginal people are very rarely genuinely consulted in the formation and functioning of a health service or health program (Lauria and Slotterback, 2021). Authentic partnership remains uncommon (Martin *et al.*, 2019). The establishment of Aboriginal Community Controlled Health Organisations (ACCHOs) allowed for Aboriginal people to create, lead, inform and undertake the implementation of health services and programs. However, the majority of non-ACCHO health services retain the hierarchy of control and decision making with non-Aboriginal people (Fogarty *et al.*, 2018a). Very few Aboriginal people are employed and retained in the non-ACCHO healthcare setting. It has been suggested that this is because of a lack of respect for the staff member’s cultural knowledge and limitations placed on educational opportunities and scope of practice (Sherwood, 2013). Not only has this “deficit discourse” approach proved to be unhelpful in terms of improving the health and well-being of Aboriginal people, there is mounting evidence that it has been detrimental to mental health and capacity to achieve autonomy in all aspects of life (Dudgeon *et al.*, 2014; Fogarty *et al.*, 2018a). It may be thought that discourse is ‘ust language’; however, research has demonstrated that it is indivisible from a person’s world view and consequent behaviour (Bourke *et al.*, 2013; Fforde *et al.*, 2013; Thomas *et al.*, 2014; Hogarth, 2017b; Fogarty *et al.*, 2018a). As a result, discourse is pivotal in resource and power inequalities. Negative discourse and discriminatory power imbalances have had a profound impact on identity formation, educational attainment and employment, as well as the health and well-being of Aboriginal people (Bourke *et al.*, 2013; Hogarth, 2017a; Fogarty *et al.*, 2018a; Dockery, 2020). The abuse of power has led to trauma, and redressing this imbalance is core to authentic partnerships, building trust and supporting recovery (Dudgeon and Walker, 2015; Brown, 2019).

Brown (2019) writes that the deficit discourse has resulted in a lack of value of the opinions and worldview of Aboriginal people. The racism and discrimination demonstrated by many non-Aboriginal people at the time of colonisation continues to permeate all facets of Australian society (Sherwood, 2013; Martin *et al.*, 2019).This impacts policies that implicitly diminish the significance of the priorities of Aboriginal Australians (Brown, 2019). The deficit discourse contributes to racism which is often internalized by Aboriginal people. Ray Mahoney wrote in The Conversation, (2020) that many Aboriginal people are reluctant to disclose their Aboriginality to health professionals for fear of receiving sub-standard service. He explained that that Aboriginal clients had longer wait times for treatment than non-Aboriginal clients and often had poorer outcomes post discharge.

## The voice for change and the implementation of an authentic partnership approach for the delivery of services

In response to the patronising and paternalistic way that health services have been delivered over many years, Aboriginal people have become united in their voice for change (Fogarty *et al.*, 2018b). The result is the implementation of the partnership approach for the delivery of services that is truly strength based in philosophy, policy and practice (Geia *et al.*, 2011; Rowley *et al.*, 2015; Durey *et al.*, 2016a). Arising from social work practice with youth in the 1990s, the core belief of the partnership strength-based approach is that all individuals have strengths and resources and that the focus of practice should, therefore, be on a person’s skills, interests and support systems (Laursen, 2003; Craven *et al.*, 2016). A strength-based approach moves the focus away from Western norms and places the emphasis on the client’s values. This has an empowering effect on well-being as self-determination is promoted (Barwick, 2004; Rowley *et al.*, 2015). According to Barwick, (2004), the basic premise is to identify what is going well, to do more of it, and to build on it. In a recent report published by the Lowitja Institute and National Centre for Indigenous Studies, Fogarty and colleagues, (2018a) outline the findings of a systematic review of Australian Aboriginal and Torres Strait Islander strengths-based health literature and conclude that they ‘are hopeful that such approaches will continue to be critically explored, developed and implemented, and that recognising the rights, culture, diversity and strengths of Australia’s First Peoples will become the norm’ (Fogarty *et al.*, 2018a: viii).

The concept of cultural competence has been examined a great deal in the literature (Cross *et al.*, 1989; Saha *et al.*, 2008; Bainbridge *et al.*, 2015). The focus has been fuelled by increasing concerns about ongoing inequalities in healthcare provision with poor health outcomes for Indigenous people throughout the world (Bainbridge *et al.*, 2015: 4). Cultural competence has been defined as ‘…a set of congruent behaviours, attitudes and policies that come together in a system, agency or amongst professionals and enables…those…to work effectively in cross-cultural situations…’ (Cross *et al.*, 1989: 28). In practical terms, cultural competence should result in a willingness of CHN to understand the perspective of the client and client’s family and situation (Bainbridge *et al.*, 2015; McCalman *et al*., 2012). It includes attitudes, communication, mutual goal setting and an understanding of the context of the client (Cross *et al.*, 1989; Bainbridge *et al.*, 2015). A vital attribute to achieve cultural competence is cultural humility (Foronda *et al.*, 2016). The concept of cultural humility gained attention in 1998 when Tervalon and Murray-Garcia purposed that it was vital to clinician practice if power imbalances were to be addressed. Cultural humility involves ongoing self-assessment and reflection on one’s own behaviours and beliefs, an openness to explore new ideas and an equitable and supportive approach to others (Tervalon, 1998; Isaacson, 2014; Foronda *et al.*, 2016). Cultural competence not only involves the CHN and client relationship but it also incorporates organisational structures. Organisational factors such as such as cost, location, wait times, appointment flexibility, open hours, and use of a home visiting service and the employment of Aboriginal people in positions of leadership and influence all effect the cultural competence of the establishment (Bainbridge *et al.*, 2015). Cultural competence is vital for reducing inequalities in health facility access and for improving health outcomes (Craven *et al.*, 2016). Being culturally competent is more than being culturally aware. It involves ‘behaviours, attitudes and policies’ that come together to effectively work across cultures (Bainbridge *et al.*, 2015: 2). As cultural competence increases so will the responsiveness of CHN and organisations to Aboriginal people (Bainbridge *et al.*, 2015).

Communication styles vary across cultures. Non-direct forms of conversation, such as ‘yarning’, are often used within Aboriginal communities (Bessarab and Ng’andu, 2010). Aboriginal people across Australia also practice a form of deep quiet listening and contemplation. The concept and practice which involves a oneness with body, spirit and the earth has become known as Dadirri which is from the languages of Aboriginal people of the Daly River region in the Northern Territory. The Dadirri approach allows for deeper discussion of sensitive issues in a way that is respectful and reduces feelings of shame, anxiety and other similar emotions that can be triggered in unsafe environments, particularly where there is trauma (Craven *et al.*, 2016; Atkinson *et al.*, 2021). However, CHN are not accustomed or trained to facilitate indirect gentle inquiry approaches (Durey *et al.*, 2016a). The emphasis for the CHN is on completing the organisational requirements of the consultation (Bessarab and Ng’andu, 2010; Lin *et al.*, 2016). This involves the asking of a list of questions to which a direct response is expected. This approach often results in Aboriginal clients feeling intimidated and subsequently withdrawing from the interaction (Durey *et al.*, 2016a; Munns *et al.*, 2017). A more acceptable form of communication which utilises an open and indirect conversational approach, whilst still allowing the CHN to obtain and share the information required, results in Aboriginal clients feeling less threatened and more willing to engage (Durey *et al.*, 2016b). Open and gentle communication is required if true partnership is to exist particularly when working with Aboriginal people who have a strong history of power imbalance and trauma (Sherwood, 2013; Splaine Wiggins, 2008).

A predominant theme of the partnership approach is mutual goal setting. Mutual goal setting involves joint discussions between the primary caregiver and the CHN which seek to highlight the strengths within the family and the desired health outcomes for the client (Eisler and Potter, 2014). Help-seeking behaviour is central to the process of setting mutual goals. Furthermore, a detailed understanding of help-seeking behaviour is relevant to assessing the physical and psychosocial contexts of Aboriginal people’s lives. Help seeking refers to the factors that lead to a person seeking assistance for a potential or actual problem (Cornally and McCarthy, 2011). The topic has been widely addressed in the academic literature in recent years (Cornally and McCarthy, 2011; Zimmerman, 2013; Ng and Lucianetti, 2016). Mostly, the literature has focused on people’s help-seeking behaviour in relation to mental health problems (Cornally and McCarthy, 2011). More recently, the concept has been applied to Indigenous people in Canada and New Zealand and Aboriginal people in Australia (Stevenson *et al.*, 2017). Help-seeking behaviour has been suitably explained by Social Cognitive Theory (Bandura, 2012). Social Cognitive Theory proposes that people learn behaviour through the observation of others (Bandura, 2012). An individual’s behaviour is influenced by their environment and personal attributes and perceptions (Ng and Lucianetti, 2016). A person’s environment is divided into the social and physical environment. The social environment encompasses the family, friends and people around the individual. In Aboriginal communities, the importance of family enhances the influence of the social environment on an individual’s behaviour (Ledogar and Fleming, 2008; Fogarty *et al.*, 2018b). In Social Cognitive Theory, the physical environment includes factors such as the availability of fresh affordable food, heating or cooling in the home and adequate housing (Bandura, 2012).

Key concepts of the theory include those of self-efficacy and collective efficacy. Self-efficacy is explained as being the individual’s ability to adopt certain behaviours. Self-efficacy is influenced by a person’s previous exposure to the desired behaviour and the belief that they have control enough to execute and maintain the desired change (Lubans *et al.*, 2012). Collective efficacy is described as the ability of a group of people to control the behaviours of individuals (Bandura, 2012). In Aboriginal culture, where family connections are highly valued, collective efficacy must be considered as a strong influence over an individual’s behaviour (Fogarty *et al.*, 2018a). Collective efficacy, or the control of one’s social circle over an individual’s behaviour, is shaped by the norms, values and expectations of the group (Bandura, 2012). The Social Cognitive approach offers the opportunity for enhanced social support through the promotion of observational learning, altering expectations and strengthening self-efficacy (Lin, 2016). The theory can be used to understand the social determinants of health and their influence on a person’s past experience with behaviour change (Thurber *et al.*, 2018).

Social Cognitive Theory can also be employed to gain a greater understanding of the behaviours and characteristics of Aboriginal primary caregivers, Aboriginal community leaders and health service providers with regard to the assessment of social and physical environments and mutual goal setting. Traditionally, health promotion activities have been focussed on individuals and their help-seeking behaviour. There has been little attention given to the significance of the social capital of the community or group (MacKian, 2003). Social capital is described as being the norms, values and resources of the wider group or community (Bandura, 2012). There is growing acknowledgement in international and Australian literature that there is a complex array of factors that interplay to influence an individual’s help seeking (Maggi *et al.*, 2010; Abubakar *et al.*, 2013; Stevenson *et al.*, 2017). Focusing attention purely on health systems and individuals for solutions to health problems ignores the influence that society has on the help-seeking behavior of an individual. The ability of social capital to influence the individual’s ehavior, or collective efficacy as it is known in Social Cognitive Theory, needs to be acknowledged if health programs are to result in positive ehavior change (Ledogar and Fleming, 2008; Stevenson *et al.*, 2017; Thurber *et al.*, 2018). Attention to the attitudes of the wider community could contribute to vital understanding of why, when and how people ehavio health facilities and adopt and maintain ehavior change (Stevenson *et al.*, 2017). In Australian Aboriginal community groups, historical encounters with government and non-government organisations have often negatively influenced the trust communities have in organisations which are governed by people outside of their own group. In order to promote a positive involvement in healthy behaviours, health organisations need to encourage the involvement of the Aboriginal community in facets of organisational structure and the management and delivery of programs (Fogarty *et al.*, 2018b).

## Conclusion

Despite this somewhat negative assessment of CHN practice with Aboriginal families regarding the health and well-being of their preschool-aged children, we are very optimistic that the authentic partnership approach to care we have outlined in this paper will be implemented widely in the near future. This positive assessment is based on the fact that Aboriginal people are now asserting themselves as leaders in all aspects of Aboriginal life, including health and welfare. This is evidenced in the most recent Closing the Gap document published by the Commonwealth Government. “The way all levels of government and Aboriginal and Torres Strait Islander representatives have come together to negotiate this National Agreement and collectively determine how we strive to close the gap demonstrates our commitment to working together through meaningful partnerships,” Minister Wyatt said (Anton *et al*., 2020).

A genuine strength-based approach to services moves away from Western norms and places the focus on the client’s values. As self-determination is promoted, well-being improves (Rowley *et al.*, 2015). When non-Aboriginal CHN understand and respect the perspective and context of Aboriginal people, an authentic working partnership can develop. It is with this authentic partnership approach to care that the health and well-being of Aboriginal children can be optimised.
